# Lymphoma-on-chip model reveals that lymph node stromal cells promote diffuse large B-cell lymphoma survival and migration

**DOI:** 10.1016/j.mtbio.2025.101544

**Published:** 2025-02-07

**Authors:** Mohammad Jouybar, Aleksandra M. Mikula, Nanouk Zuidmeer, Tanja Konijn, A. Vera de Jonge, Henk P. Roest, Tuna Mutis, Luc J.W. van der Laan, Reina E. Mebius, Jaap M.J. den Toonder, Charlotte M. de Winde

**Affiliations:** a Microsystems, Eindhoven University of Technology, Eindhoven, the Netherlands; b Institute for Complex Molecular Systems, Eindhoven, the Netherlands; c Department of Molecular Cell Biology, Amsterdam UMC Location VU, Amsterdam, the Netherlands; d Amsterdam Institute for Immunology and Infectious Diseases, Amsterdam, the Netherlands; e Cancer Center Amsterdam, Cancer Biology and Immunology, Amsterdam, the Netherlands; f Department of Hematology, Amsterdam UMC Location VU, Amsterdam, the Netherlands; g Erasmus MC Transplant Institute, University Medical Center Rotterdam, Department of Surgery, Rotterdam, the Netherlands

**Keywords:** Lymphoma-on-Chip, Lymphatic vessel, Vessel-on-Chip, Tumor microenvironment, Diffuse large B-Cell lymphoma, Lymph node, Lymph node stromal cells

## Abstract

Diffuse large B-cell lymphoma (DLBCL) is the most common aggressive B-cell non-Hodgkin lymphoma, often developing resistance to current treatments. Development and testing of new therapies is hampered by lack of good *in vivo* and *in vitro* models mimicking human disease. Here, we developed a lymphoma-on-chip model to investigate the tumor-supportive roles of lymph node stromal cells (LNSCs) – fibroblastic reticular cells (FRCs) and lymphatic endothelial cells (LECs) – in the DLBCL microenvironment. The model includes a tubular vessel lined with LECs surrounded by a hydrogel with DLBCL cells and FRCs. Our findings reveal that FRCs promote DLBCL survival and facilitate tumor cell migration towards lymphatic vessels. Moreover, we demonstrate that DLBCL cells increase permeability of lymphatic vessels, which is further enhanced in presence of FRCs. This lymphoma-on-chip model reveals the important role of LNSCs in DLBCL progression, and offers an innovative tool to study the DLBCL microenvironment and test potential therapeutic targets to improve patient outcomes.

## Highlights

• Human lymphoma-on-chip model with lymph node stromal cells (LNSCs) was developed.
• Lymphatic vessel is surrounded by hydrogel with lymphoma cells and fibroblasts.
• Fibroblasts promote lymphoma cell survival and directional migration.
• Lymphoma cells enhance lymphatic vessel permeability.
• This model provides *in vivo*-like features enabling future drug targeting studies.

## Graphical abstract

**Figure:**
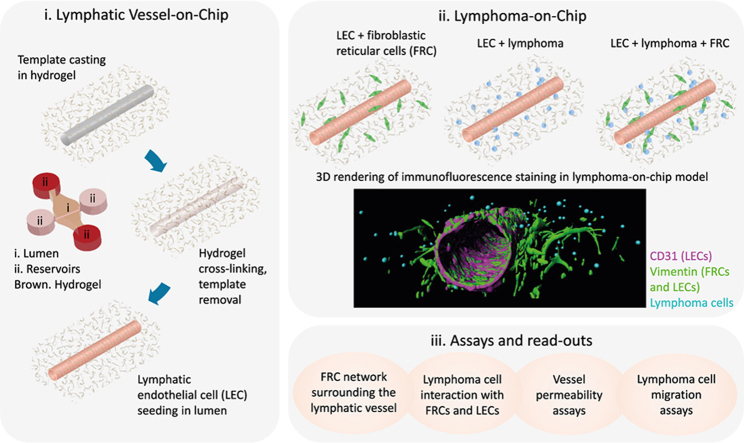

## 1 Introduction

Diffuse large B-cell lymphoma (DLBCL) is the most common aggressive type of B-cell non-Hodgkin lymphoma, accounting for one third of all lymphoma cases with a 5-year survival rate of about 60 % [1]. DLBCL patients are treated with combined immuno-chemotherapy (R-CHOP), but still many patients relapse since they develop therapy resistance within the first 2–3 years after diagnosis, making DLBCL an incurable disease for about 40 % of patients. In recent years, new treatment options are being explored, such as cellular and antibody therapies [2]. However, DLBCL is a molecular, biological and clinical heterogenous disease, underscoring the relevance of patient stratification to enable personalized medicine treatment strategies.

DLBCL arises from genetically altered B-lymphocytes in lymph node follicles upon activation of the adaptive immune response [3]. Lymph nodes have a highly structured tissue architecture, which is controlled by lymph node stromal cells (LNSCs), including fibroblastic reticular cells (FRCs) and lymphatic endothelial cells (LECs) [4,5]. LNSCs produce and secrete cytokines and chemokines that respectively promote immune cell survival and migration. Furthermore, FRCs form an interconnected reticular network that serves as migration route for immune cells through the lymph node.

DLBCL tumors do not only contain tumor cells, but also house immune and stromal cells, together forming the DLBCL microenvironment. In DLBCL, different stromal transcriptome signatures have been described that correlate with clinical outcome [[6], [7], [8]]. More recently, functional studies have been performed showing that FRCs within the DLBCL microenvironment gain a tumor-activated phenotype, which suppresses the anti-tumor immune response [9]. However, the direct role of FRCs as well as LECs in promoting DLBCL progression and as such contributing to a DLBCL-supporting niche within lymph nodes is still unclear. We hypothesize that DLBCL-activated LNSCs secrete soluble factors promoting tumor cell survival, and that the FRC network is being hijacked as dissemination route by DLBCL tumor cells to eventually intravasate into lymphatic vessels and metastasize to distant sites. Insight in the tumor-supportive functions of LNSCs in the DLBCL microenvironment paves the way for designing new therapeutic strategies for DLBCL patients.

Functional studies on the contribution of interactions between DLBCL cells and LNSCs to tumor progression and dissemination are hampered by the lack of suitable *in vivo* and *in vitro* models mimicking the human DLBCL microenvironment. Two DLBCL mouse models spontaneously develop disease upon aging [10,11], resulting in long and expensive experiments, but above all do not fully resemble human DLBCL nor capture the heterogeneity found amongst DLBCL patients. The lymph node microenvironment is a complex system, difficult to mimic *in vitro*, due to presence of different cell types and the classical architecture formed by the FRC network and lymphatic vessels throughout the tissue. As an alternative, simple two-dimensional *in vitro* models have been used, such as feeder layers of fibroblasts to mimic cytotoxic drug resistance of chronic lymphocytic leukemia (CLL) [12], or transwell set-ups to investigate the effect of soluble factors on DLBCL-FRC crosstalk [9]. In recent years, three-dimensional (3D) models to study the role of FRCs in B-cell malignancies and to perform drug testing have been exploited, such as spheroids for CLL [13] and microfluidic alginate microspheres for follicular lymphoma (FL) [14]. However, these are static models which lack tissue organization and lymphatics to study DLBCL migration and dissemination.

Organ-on-chip models can provide these features and have the potential to emulate human disease better than conventional models [15]. Furthermore, cancer-on-chip models are exploited to study tumor cell migration and metastasis [16]. A 3D microvascular model for DLBCL has been reported consisting of a hydrogel with tumor and immune cells and an endothelialized lumen used for delivering drugs to the tumor model [17]. However, this 3D DLBCL model does not include FRCs nor lymphatics, which are both important in the tumor microenvironment and cancer metastasis [9,18] and can be incorporated and functionally studied in *in vitro* systems [[19], [20], [21]]. To investigate how both FRCs and LECs contribute to shaping a DLBCL-promoting environment, we here generated an innovative lymphoma-on-chip model including FRCs and a lymphatic vessel. We used primary FRCs and LECs derived from human lymph nodes [19] and created a lymphatic vessel by a template casting method. The hydrogel containing FRCs and DLBCL cells was cast around a needle, which was extracted after crosslinking the hydrogel resulting in a tubular microchannel within the hydrogel [22] which we lined with LECs to generate a lymphatic vessel. Next, we used this model to investigate the tumor-supportive roles of FRCs and LECs in DLBCL survival and migration.

## 2 Results

### 2.1 Lymph node stromal cells are present in DLBCL tumors

Lymph nodes consist of different functional areas, like B- and T-cell zones, important for initiating an efficient immune response. This characteristic tissue architecture is shaped by LNSCs, including FRCs and LECs, which can be characterized by expression of Vimentin (Fig. 1A), a cytoskeletal protein expressed in cells of mesenchymal origin [23]. In contrast, in DLBCL, this characteristic lymph node architecture is completely disrupted when DLBCL tumor cells populate the tissue [9]. However, as also reported by others [9], Vimentin^+^ LNSCs are still present in between the tumor cells (Fig. 1A), and this can be mimicked *in vitro* by co-culture of DLBCL tumor cells (human DLBCL cell line OCI-Ly18) and primary human FRCs in a collagen hydrogel, where the Vimentin^+^ FRCs are localized within and around clusters of DLBCL cells (Fig. 1B). Thus, LNSCs remain present when a DLBCL tumor develops within lymph nodes, and are localized between the tumor cells, which can be mimicked *in vitro*.

**Fig. 1:**
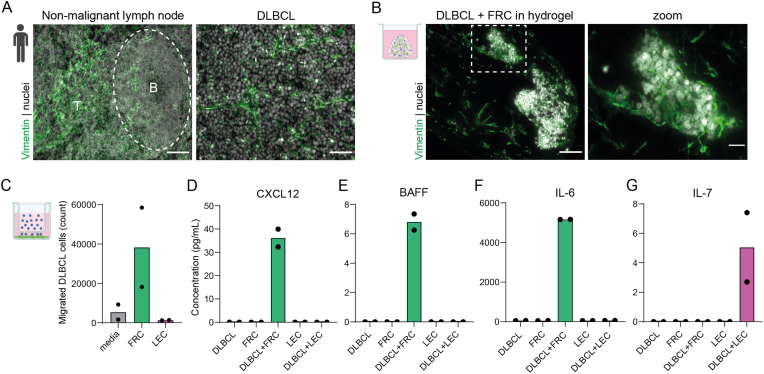
Lymph node stromal cells are present in DLBCL tumors, attract DLBCL tumor cells and produce migration and survival factors. **A.** Immunofluorescence staining of Vimentin (green) in human non-malignant (left) and diffuse large B-cell lymphoma (DLBCL)-bearing lymph node (right). Nuclei are shown in white. B-cell follicle is indicated by dashed line; T = T-cell zone. Scale bars are 100 μm. **B.** Immunofluorescence staining of Vimentin (green) in fibroblastic reticular cells (FRCs) in co-culture with DLBCL cells in a hydrogel for 10 days. Nuclei are shown in white. Scale bars are 100 (left) and 25 (right) microns. **C.** Number of migrated DLBCL cells towards media (grey), FRC (green) or lymphatic endothelial cells (LEC; magenta) in a transwell migration assay. DLBCL cells were embedded in a collagen type I hydrogel (2.5 mg/mL) and allowed to migrate for 5 days. Data shown as mean. Individual data points represent two different donors. **D-G.** Concentration of chemokine CXCL12 (**D**) and survival factors BAFF (**E**), IL-6 (**F**) and IL-7 (**G**) in supernatant taken from the bottom well of the transwell migration assay shown in C. Data shown as mean. Individual data points represent two different donors. (For interpretation of the references to color in this figure legend, the reader is referred to the Web version of this article.)

### 2.2 Lymph node stromal cells attract DLBCL tumor cells and produce migration and survival factors

In homeostasis and during an immune response, FRCs and LECs provide survival factors to B- and T-lymphocytes, and the FRC network serves as a migration route for immune cells [4,5]. We hypothesize that DLBCL tumor cells hijack the immune-supportive functions of LNSCs to promote their survival and migration. To test this, we performed a transwell migration assay of DLBCL cells embedded in a collagen hydrogel which were allowed to migrate for 5 days towards FRCs or LECs, or their respective culture media as control. DLBCL cells migrated towards FRCs, but not towards LECs (Fig. 1C). To investigate the mechanism underlying tumor cell migration, we measured levels of chemokines and cytokines, soluble factors produced by LNSCs which can drive migration and survival respectively, in the culture media after DLBCL migration. We found that FRCs produced and secreted the chemokine CXCL12 when DLBCL cells were present, but not in the absence of DLBCL cells (Fig. 1D). Moreover, both FRCs and LECs produced survival factors, respectively BAFF and IL-6, and IL-7, only when DLBCL cells were present (Fig. 1E–G). Thus, LNSCs can promote tumor cell survival, and FRCs, but not LECs, produce and secrete chemokines attracting DLBCL tumor cells, suggesting tumor-supportive roles for LNSCs in the DLBCL tumor microenvironment.

### 2.3 Generation of the lymphoma-on-chip model with a lymphatic vessel and FRC network

**Fig. 2:**
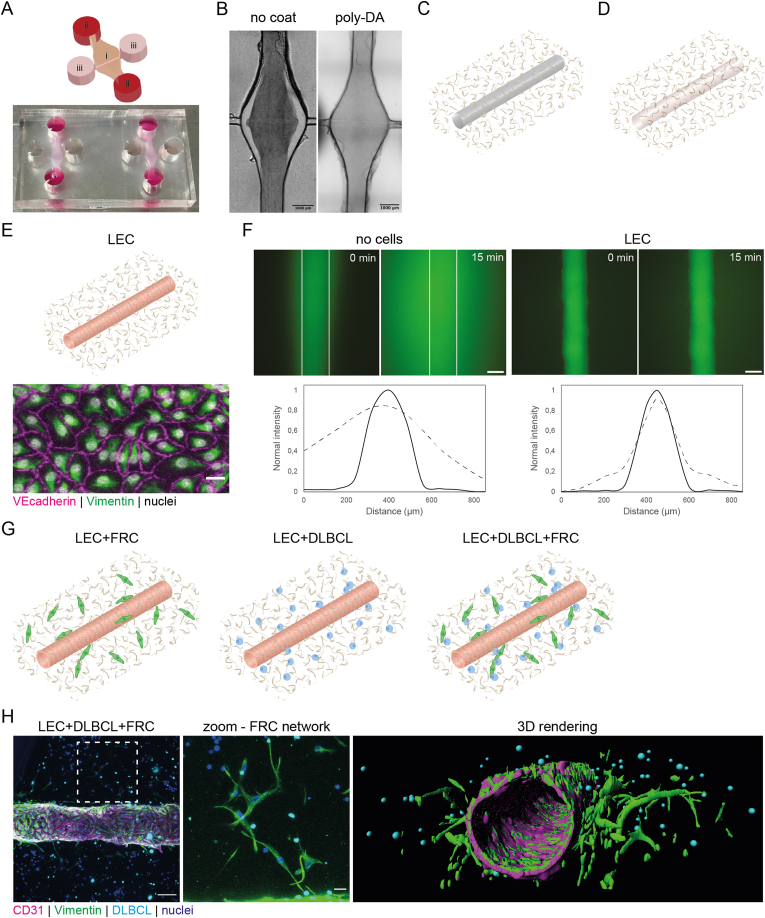
Generation of the lymphoma-on-chip model with a lymphatic vessel and FRC network. **A.** Top: schematic of chip design. i = lumen, ii = reservoirs with FRC media (red), iii = reservoirs with LEC media (pink), brown = hydrogel. Bottom: image of chips in culture. **B.** Brightfield overview images of collagen type I hydrogel (5 mg/mL) in the chip coated with polydopamine (right) or without coating (left). **C.** Schematic of needle (diameter 160 μm) through hydrogel. **D.** Schematic of polymerized and crosslinked hydrogel after the needle is extracted leaving a lumen. **E.** Top: schematic of lumen in hydrogel lined with LECs. Bottom: immunofluorescence staining of VE-cadherin (magenta) and Vimentin (green) of the LEC-lined lumen after 4 days of culture. Nuclei are shown in white. Scale bar is 25 μm. **F.** Top: fluorescence images of lumen with no cells (left panel) or lined with LECs (right panel) at 0 and 15 min after adding dextran Texas-Red (70 kDa; green). Scale bars are 100 μm. Bottom: quantification of diffusion shown in the top panels at 0 min (solid line) and 15 min (dashed line). **G.** Schematic of hydrogel lined with LECs surrounded by a hydrogel containing only FRCs (green; left), only DLBCL cells (blue; middle), or both (right). **H.** Left: immunofluorescence staining of CD31 (labelling LECs; magenta) and Vimentin (labelling FRCs as well as LECs; green) in a chip with CellTracker Deep Red-labelled DLBCL cells (cyan) after 7 days of culture. Nuclei are shown in blue. Scale bar is 100 μm. Middle: zoom of left image, indicated by dashed line, showing the FRC network in hydrogel. Scale bar is 25 μm. Right: 3D rendering of the image shown in the left panel. See also Supplementary movie 1. (For interpretation of the references to color in this figure legend, the reader is referred to the Web version of this article.)

A transwell system is not a truly physiological representation of a DLBCL tumor as the cells are separated and LNSCs are not in the same 3D environment as the tumor cells. A collagen hydrogel with FRCs and DLBCL cells as presented in Fig. 1B does provide a 3D environment, however, this lacks a lymphatic vessel that enables to investigate if promoting migration drives dissemination of DLBCL tumor cells. Organ-on-chip devices have the potential to emulate human disease better than conventional models [15,16], and although several lymph node and lymphoma-on-chip models have been published [17,24,25], these all lack lymphatic vessels and FRCs. To investigate the role of FRCs and LECs in the DLBCL microenvironment, we here generated a lymphoma-on-chip model that includes a lymphatic vessel, as well as FRCs together with DLBCL tumor cells in a collagen hydrogel surrounding the vessel (Fig. 2).

Our lymphoma-on-chip model includes a tubular lumen lined with LECs forming a lymphatic vessel, surrounded by a collagen hydrogel that includes DLBCL cells and/or FRCs. The lymphatic vessel is connected to LEC media reservoirs, and two lateral reservoirs enable media refreshment for FRCs and DLBCL tumor cells in the hydrogel (Fig. 2A). The chip was made from polydimethylsiloxane (PDMS) and has a chamber to contain the collagen hydrogel. The mold for making this chamber was fabricated using stereolithography (SLA) 3D-printing method [26,27]. The PDMS layer was bonded to a glass slide. The reservoirs were 5 mm both in diameter and height, containing enough cell culture media to avoid excessive evaporation during overnight incubation. Coating of PDMS with polydopamine was required for bonding the collagen to the surrounding walls of the chamber to counteract hydrogel contraction during long-term cell culture, especially in the presence of contractile FRCs (Fig. 2B) [28,29]. To form the vessel lumen, a needle with a diameter of 160 μm, mimicking the *in vivo* lymphatic vessel diameter [30], was inserted in the chip prior to adding the collagen hydrogel to the chamber (Fig. 2C). Following polymerization of the collagen, the needle was extracted, resulting in a perfusable 3-mm-long tubular lumen (Fig. 2D). The lumen was then seeded with LECs, which lined the lumen forming an intact lymphatic vessel expressing VE-cadherin^+^ tight junctions (Fig. 2E). To assess the permeability of the vessel, dextran Texas-Red (70 kDa) solution was flown into the channel. Hardly any diffusion from the lumen into the collagen was observed when LECs lined the lumen in comparison to a lumen without LECs, confirming the integrity of the vessel (Fig. 2F). In this study, three different experimental conditions were tested by including different cell types in the collagen hydrogel, namely FRCs or DLBCL cells alone, or FRCs and DLBCL cells together (Fig. 2G). We use collagen type I because it is one of the natural hydrogels commonly used in this type of model [16,[31], [32], [33]]. The structure of collagen type I is well-characterized, featuring a fibrillar arrangement with random porosity. We used a concentration of 5 mg/mL which provided the most stable gel throughout the culture period with minimal structural changes. When preparing the hydrogel, we mix single cell suspensions of FRCs and/or DLBCL cells into the hydrogel and subsequently transfer it to the chip, so the cells should be randomly distributed. Within the hydrogel, FRCs elongate and subsequently proliferate. Images of our lymphoma-on-chip model with all three cell types present confirm that LECs form a tubular lymphatic vessel with FRCs forming a network within the collagen hydrogel with DLBCL cells attached (Fig. 2H and Supplementary movie 1), as seen in human (DLBCL-bearing) lymph nodes (Fig. 1A). We observe intact cell morphology and nuclei of all cell types and throughout the whole hydrogel (Figure S1), indicating that cell viability is maintained in our model by continuous supply of fresh nutrients via the reservoirs and the LEC-lined tubular lumen.

### 2.4 FRCs promote DLBCL tumor cell survival

**Fig. 3:**
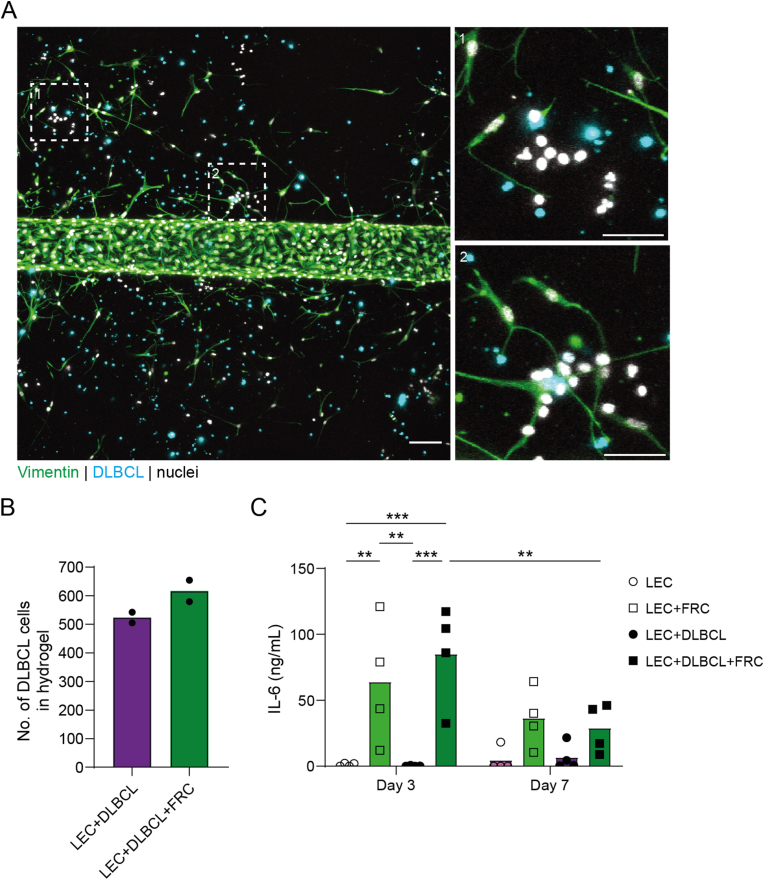
FRCs promote DLBCL tumor cell survival. **A.** Immunofluorescence staining of Vimentin (green) in the chip after 7 days of culture containing LECs, FRCs and DLBCL cells (cyan). Nuclei are shown in white. Scale bars are 100 μm (overview image) and 50 μm (zooms). Magnified images show clusters of tumor cells in close proximity to FRCs. **B.** Number of CellTracker Deep Red-labelled DLBCL cells in hydrogel in chip without (purple) and with (green) FRCs. Data shown as mean. Individual data points represent different chips with LECs and FRCs from different donors. **C.** Concentration of interleukin-6 (IL-6; in ng/mL) at day 3 and day 7 for different culture conditions including only LEC (magenta bar, open circles), LEC + FRC (light green bar, open squares), LEC + DLBCL (purple bar, closed circles) and LEC + DLBCL + FRC (dark green bar, closed squares). Data shown as mean. Individual data points represent different chips with LECs and FRCs from different donors. Two-way ANOVA with Tukey’s multiple comparisons; ∗∗*p* < 0.01, ∗∗∗*p* < 0.001. (For interpretation of the references to color in this figure legend, the reader is referred to the Web version of this article.)

Having our lymphoma-on-chip model set up, we first studied if LNSCs promote survival of DLBCL tumor cells as indicated by the observed production of survival factors by FRCs and LECs in presence of DLBCL cells (Fig. 1E–F). Within the collagen hydrogel, we observed clusters of nuclei in the hydrogel in close proximity of FRCs (Fig. 3A). These nuclei were not associated with Vimentin staining, as such indicating that these are DLBCL cells, in which the CellTracker Deep Red labelling was decreased upon proliferation. Although not significant, we observed a trend towards an increased number of CellTracker Deep Red-labelled DLBCL cells in the hydrogel in presence of FRCs compared to chips without FRCs (Fig. 3B). Interestingly, we did not detect survival factors IL-7 and BAFF, nor the chemokine CXCL12 in the media harvested from the lumen and respective media reservoirs. We speculate that these factors are being produced, and subsequently being taken up and used by the DLBCL cells in the hydrogel, and are therefore not detectable in the culture media. Notably, we did detect the lymphoma survival factor IL-6 [34,35] in the lumen and associated reservoirs of the lymphoma-on-chip model only when FRCs were present in the hydrogel and LECs in the lumen (Fig. 3C). In presence of DLBCL cells in the hydrogel in addition to FRCs, and with LECs in the lumen, a trend towards more IL-6 secretion was observed at day 3. This is in line with a similar model for head and neck cancer [33], where it was shown that co-culture of fibroblasts with LECs results in a trend towards higher IL-6 levels compared to co-culture of fibroblasts with head and neck tumor cells, and IL-6 levels further increased when all three cell types were present. However, at day 7, we observed that IL-6 levels were significantly decreased in the condition with all three cell types present (Fig. 3C), suggesting that the produced IL-6 is being used by the increased number of DLBCL cells to promote their survival and as such lower IL-6 levels are measured in the culture media over time. These data suggest that FRCs promote survival of DLBCL tumor cells by secreting IL-6. Of note, there was also a trend towards lower IL-6 levels in absence of DLBCL tumor cells at day 7 (Fig. 3C). This could be attributed to IL-6 use by FRCs and LECs, which also express IL-6R, although to a lower extent than the DLBCL tumor cells [36].

### 2.5 FRCs promote DLBCL-mediated LEC barrier loss and DLBCL cell migration

**Fig. 4:**
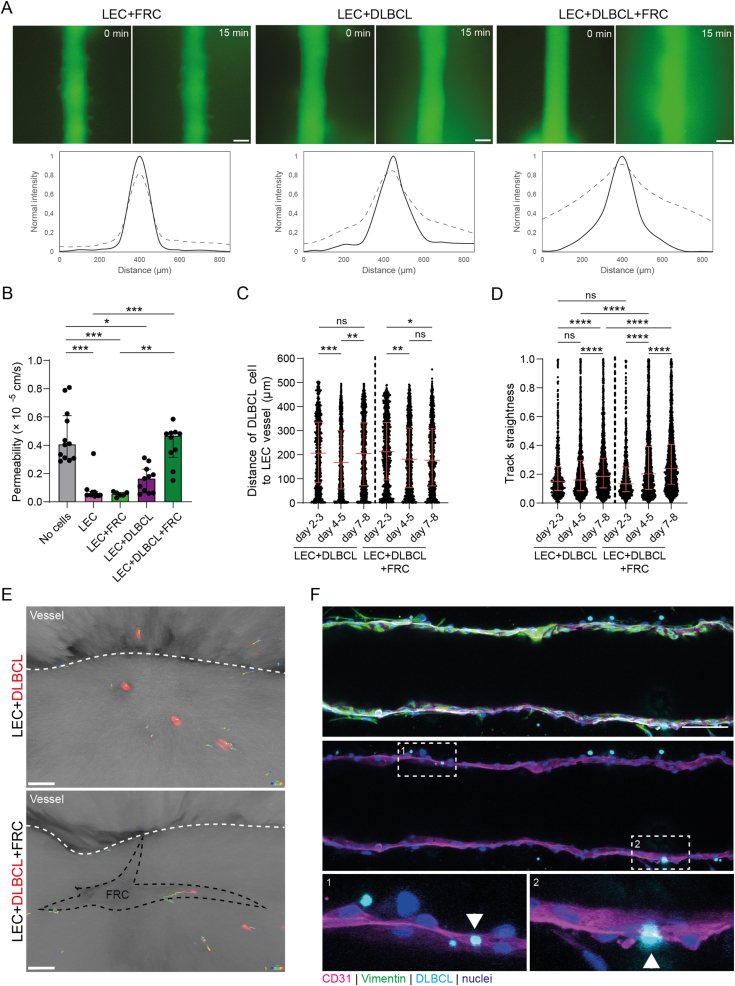
FRCs promote DLBCL-mediated LEC barrier loss and DLBCL cell migration. **A.** Top: fluorescence images of chip with LEC + FRC (left panel), LEC + DLBCL (middle panel) or LEC + DLBCL + FRC (right panel) at 0 and 15 min after adding dextran Texas-Red (70 kDa; green). Scale bars are 100 μm. Bottom: quantification of diffusion shown in the top panels at 0 min (solid line) and 15 min (dashed line). **B.** Quantification of lumen permeability based on dextran diffusion data as shown in Fig. 2, Fig. 4. Data shown as median with interquartile range of measurements performed on chips with two different lymph node donors. Individual datapoints represent different lines along which the lumen permeability is measured (N = 5–7 lines per chip). Kruskal-Wallis test with Dunn’s multiple comparisons; ∗*p* < 0.05, ∗∗*p* < 0.01, ∗∗∗*p* < 0.001. **C.** Distance (in microns) of CellTracker Deep Red-labelled DLBCL cells in hydrogel to the lymphatic vessel at different time points in LEC + DLBCL (left) or LEC + DLBCL + FRC (right) chips. Data shown as median with interquartile range of measurements performed on chips with two different lymph node donors. Individual datapoints represent different DLBCL cells. Kruskal-Wallis test with Dunn’s multiple comparisons; ∗*p* < 0.05, ∗∗*p* < 0.01, ∗∗∗*p* < 0.001, ns = not significant. **D.** Track straightness of CellTracker Deep Red-labelled DLBCL cells in hydrogel at different time points in LEC + DLBCL (left) or LEC + DLBCL + FRC (right) chips. Data shown as median with interquartile range of measurements performed on chips with two different lymph node donors. Individual datapoints represent different DLBCL cells. Kruskal-Wallis test with Dunn’s multiple comparisons; ∗∗∗∗*p* < 0.0001, ns = not significant. **E.** Endpoint images of time-lapse imaging of CellTracker Deep Red-labelled DLBCL cells (red) in hydrogel in LEC + DLBCL (top) and LEC + DLBCL + FRC (bottom) chips. The lymphatic vessel is located at the top of the images as indicated by the white dashed line. Outline of FRC is indicated by the dashed black line. Tracks of individual DLBCL cells over time are shown in rainbow colors from blue to red. Scale bars are 25 μm. See also Supplementary movies 2 and 3. **F.** Immunofluorescence staining of CD31 (magenta) and Vimentin (green) in a chip after 7 days of culture with LECs, FRCs and DLBCL cells (cyan). Nuclei are shown in blue. Scale bar is 100 μm. Arrows in magnified images point to intravasating DLBCL cells. (For interpretation of the references to color in this figure legend, the reader is referred to the Web version of this article.)

Next, we used our lymphoma-on-chip model to investigate whether LNSCs play a role in the dissemination of DLBCL cells. For solid tumors, it has been reported that tumor cells as well as cancer-associated fibroblasts (CAFs) can increase the permeability of lymphatic vessels to aid tumor cell intravasation and metastasis [18,[37], [38], [39]]. We observed that DLBCL cells increase the permeability of the lymphatic vessel, which was even further enhanced when FRCs were present (Fig. 4A–B). This increased lymphatic vessel permeability was driven by presence of DLBCL cells, as FRCs alone did not influence its permeability (Fig. 4A–B).

Next, we investigated DLBCL tumor cell migration in our lymphoma-on-chip model and the role of LNSCs herein. First, we measured the distance of DLBCL cells to the lymphatic vessel over time. When only DLBCL cells were present in the hydrogel, the distance to the lymphatic vessel decreased from day 2–3 to day 4–5, but increased again at day 7–8 (Fig. 4C). However, when both DLBCL cells and FRCs were co-seeded in the hydrogel, the distance of tumor cells to the lymphatic vessel further decreased at day 7–8 (Fig. 4C). These data suggest that LECs can attract DLBCL cells, but that the network of FRCs (Fig. 2H) is required to further direct the DLBCL cells towards the lymphatic vessel. Indeed, the presence of FRCs increases the track straightness of CellTracker Deep Red-labelled DLBCL cells, indicating that FRCs promote directed migration of DLBCL cells (Fig. 4D–E and Supplementary movies 2-3). Moreover, we observed interaction of DLBCL cells with the lymphatic vessel and captured DLBCL cells at the moment of intravasating into the lymphatic vessel from the hydrogel through the LECs lining the lumen (Fig. 4F). In summary, the results obtained with this lymphoma-on-chip model, including FRCs and a tubular lymphatic vessel, indicate that LNSCs, and specifically FRCs, promote DLBCL survival and migration.

Supplementary data related to this article can be found online at https://doi.org/10.1016/j.mtbio.2025.101544

The following are the Supplementary data related to this article:

Video 1 Video 1

Video 2 Video 2

Video 3 Video 3

Video 4 Video 4

Video 5 Video 5

## 3 Discussion

An important role for LNSCs in the DLBCL microenvironment has become apparent in recent years [[7], [8], [9]], but experimental models for functional studies mimicking the human DLBCL microenvironment were lacking. Here, we present an innovative human lymphoma-on-chip model including DLBCL tumor cells and FRCs in a hydrogel surrounding a tubular lymphatic vessel generated using the needle template casting method. Using this lymphoma-on-chip model, we show that LNSCs, and especially FRCs, promote survival and migration of DLBCL tumor cells, as such contributing to lymphoma progression.

Template casting is one of the useful methods for including microchannels within hydrogels in organ-on-chip devices [16,31]. Various types of templates have been used in this method including PDMS rods and needles [32,40,41]. Here, we used a needle with a diameter of 160 μm for generating a tubular microchannel within a collagen hydrogel. This method is a robust and relatively simple templating method enabling integration of tubular microchannels within a hydrogel, mimicking tubular structures *in vivo*, like microvessels found within tissues. The tubular geometry influences the morphology and orientation of endothelial cells lining the vessel [42,43]. Nevertheless, this method includes a manual needle extraction step which is not favorable for scaling up towards higher throughput models. Needle removal should be done carefully to avoid disrupting the hydrogel, which is more prevalent with lower gel concentrations. We used a collagen concentration of 5 mg/mL in our experiments, and the template casting technique has been successfully utilized with a collagen concentration as low as 2.5 mg/mL [32]. Moreover, this needle template casting technique is limited to a straight luminal channel. Alternative templating methods can potentially solve these issues. For realizing more intricate tubular geometries, sacrificial materials have been 3D-printed as templates for obtaining tubular channels embedded in hydrogel or in cured polymer [[44], [45], [46], [47], [48]]. For example, carbohydrate glass fibers were printed as sacrificial molds to generate perfusable patterned tubular networks with complex shapes [45,47,49]. In other studies, bioprinting inks, such as pluronic F-127 and gelatin, were used for 3D printing the sacrificial mold [46,48]. For example, Kolesky et al. fabricated a thick vascularized tissue including a network of endothelialized tubular channels enabling long-term perfusion [46]. In a recent study, Zhang et al. fabricated a system of two parallel lumens using 3D printing of gelatin as sacrificial mold [48]. While these techniques have greatly contributed to advancing our ability to create tubular channels for organ-on-chip applications, yet more sophisticated templating techniques can pave the way towards more robust and higher throughput models.

To promote anchoring of the hydrogel to the chamber walls of the chip, polydopamine coating was required to counteract gel contraction and prevent delamination of the gel from the walls [28,29]. This is crucial when the culture includes contractile cells, such as fibroblasts, which elongate and reshape the matrix significantly [50]. In similar studies, glutaraldehyde solution was used to solve this issue [32,40], but we chose polydopamine as this is less toxic for cells [28,29]. While the presence of polydopamine may not be physiological for our application, thorough washing of the chamber with PBS prior to hydrogel introduction removes the excess polydopamine solution. Additionally, the vessel channel and cells within the matrix are embedded in the hydrogel, avoiding direct contact with the surrounding chamber walls thus minimizing the potential impact of polydopamine on our tests.

By seeding and culturing LECs in the microchannel, we successfully generated a lymphatic vessel with barrier function, as determined by measuring dextran diffusion showing low permeability in line with previous publications [37,51]. While DLBCL cells alone increased permeability of the lymphatic vessel, this was further enhanced by presence of FRCs. However, the molecular mechanisms underlying increased lymphatic vessel permeability by DLBCL cells, the role of FRCs herein, and the effect on DLBCL progression remain to be investigated. It was shown in a vessel-on-chip model that breast cancer-derived cancer-associated fibroblasts (CAFs) drastically increased the permeability of the lymphatic vessel [37]. When CAFs were present, large holes were observed in the vessel, as well as increased levels of growth factors, chemokine IL-8/CXCL8 and cytokine IL-6 compared to presence of normal fibroblasts. Vessel permeability was partly restored by blocking the IL-6 receptor (IL-6R), and fully by neutralizing IL-6 using antibodies [37]. In DLBCL, high IL-6 serum levels correlate with decreased patient survival [52]. In our lymphoma-on-chip model, we detected IL-6 in the luminal supernatant when FRCs were present in the hydrogel, and IL-6 levels increased when both FRCs and DLBCL cells were present, supporting the hypothesis that FRCs are the source of high IL-6 levels in DLBCL. This is in line with published RNA sequencing data of FRCs showing activation of the IL-6 signaling pathway after co-culture with DLBCL cells [9]. To gain further insight in the effects of FRC-driven IL-6 signalling in DLBCL tumor cells, the downstream molecular pathways, for example phosphorylation of STAT3, could be investigated in future studies.

In our study, FRCs promoted directional migration of DLBCL cells towards the lymphatic vessel. The published RNA sequencing data of FRCs show activation of chemokine signaling after co-culture with DLBCL cells [9], and in follicular lymphoma (FL), another type of B-cell lymphoma, CXCL12 production by FL-CAFs was shown to promote tumor cell migration [53]. Indeed, we found increased expression of the chemokine CXCL12 by FRCs in a DLBCL transwell migration assay, indicating that this chemokine may play a role in DLBCL attraction and migration by FRCs, but the molecular mechanism and factors driving DLBCL dissemination remain to be further investigated. In a cancer-on-chip model of head and neck cancer including a lymphatic vessel increased migration of tumor cells was observed in presence of CAFs, and not in presence of normal fibroblasts [33]. Blocking IL-6 or IL-6R did not decrease viability nor migration of the cancer cells, whereas inhibiting macrophage migration inhibitory factor (MIF) did decrease tumor cell migration, but not viability. IL-8/CXCL8 can be produced by various cell types, including fibroblasts and endothelial cells, and is mainly known as a chemoattractant for neutrophils, playing an important role in the response to infection and tissue damage [54]. The role of IL-8/CXCL8 in the tumor microenvironment is less understood, although it has been suggested that it promotes tumor cell proliferation and migration [54]. These studies indicate that tumor cell survival and migration are controlled by various factors. As such, blocking one factor is most likely not sufficient, and testing combination therapies will be necessary to find the ideal therapeutic strategy for cancer patients.

The lymphoma-on-chip model presented here is a relatively simple model that can be developed to further enhance its physiological representation of the DLBCL microenvironment. Our model allows for establishing a pressure difference via application of a constant fluid pressure from the hydrogel reservoirs to mimic the lymphatic vessel drainage and/or propulsion [37]. This outlines an outlook for enhancing the system's capabilities by mimicking *in vivo* conditions through the application of differential hydrostatic pressures in the existing media reservoirs. Currently, our system operates in a static state, where constant hydrostatic pressure, derived from the media in the reservoirs, is applied to the system. During media refreshment, flow through the lymphatic vessel is induced by introducing media into one reservoir, allowing it to flow to the other reservoir via capillary action driven by hydrostatic pressure. The maximum shear stress experienced by the cells is less than 0.3 Pa, however, this is not continuous and is not in physiological range. Others in the literature, call this a dynamic system [37], but we believe this is still a static system. A second lumen can be integrated [22] to be lined with blood endothelial cells, as both lymph and blood vessels are important for lymph node tissue function. Within DLBCL tumors, angiogenesis results in increased microvessel density correlating with inferior outcome for DLBCL patients [55]. Furthermore, FRC-mediated loss of functionality of high endothelial venules (HEVs), the lymph node's entry ports for immune cells formed by blood endothelial cells, impairs immune cell trafficking into DLBCL tumors [56], which most likely impairs the anti-tumor immune response and the effect of immunotherapy. As such, incorporating a blood vessel in our lymphoma-on-chip model will open the possibility to study preferential routes of DLBCL dissemination and test therapeutic strategies inhibiting angiogenesis [57,58]. Of note, in such a dual-vessel-on-chip model, different flow modules should be utilized to apply the specialized flow rates of lymphatic and blood vessels [59].

We here used a collagen type I hydrogel, which is a natural hydrogel commonly used in this type of model providing stability throughout the culture period [16,31,[60], [61], [62]]. In addition to collagen, other hydrogels such as fibrin, Matrigel, or combinations with varying concentrations are frequently employed [63,64]. However, none of these hydrogels fully capture the *in vivo* extracellular matrix (ECM), which is a highly complex component of the microenvironment, comprising various types of proteins with distinct characteristics [65]. *In vitro* models often simplify the ECM to one or two types of hydrogels, depending on the specific research question. This simplification helps isolate and study particular aspects of the microenvironment by omitting other components. For instance, studying cellular migration over longer periods often necessitates collagen due to its fibrillar structure and high stability. Conversely, fibrin is better suited for mimicking vascularization and studying angiogenesis [16,63,66]. FRCs are the main producers of ECM within lymph nodes [67]. In DLBCL, changes in FRC-derived ECM signatures are reported [7,9], as well as increased abundance of an ECM-producing FRC subset [9]. Moreover, the stiffness of the microenvironment determines growth of DLBCL tumor cells [68]. As such, changing the hydrogel in lymphoma-on-chip models to more closely resemble the ECM characteristics found in DLBCL tumors is a challenge to take on in future studies.

To integrate the lymphoma-on-chip model into an industrial process enabling commercialization and use in a (pre-)clinical setting, a higher throughput version of the presented model is necessary. A similar model, including several chips, was previously fabricated using templating series of magnetic-head PDMS rods which enabled the simultaneous template extraction using a magnet [40]. In our model, multiple needle templates can be connected, allowing simultaneous insertion and extraction. Furthermore, the dimensions and materials of the platform are crucial for scaling up the model and commercialization. The reservoirs and tissue chamber dimensions as well as the chip-to-chip distance should be designed to be compatible with existing standard platforms and apparatus, such as multi-well plates and commercially available multi-pipetting robots [69,70]. Additionally, alternative materials to PDMS, such as plastics and glass, should be considered because of the high absorption of small molecules, which is not suitable for therapeutic studies [71,72]. This should not pose a significant hurdle, as the chip's dimensions are suitable for other fabrication methods using various materials, making the device adaptable towards higher throughput versions. This system features a relatively simple design, which enhances its reproducibility. Furthermore, similar systems have already been developed and tested for different applications [32,40], demonstrating the robustness of the concept. By reproducing this model specifically for our research question, we provide additional evidence supporting its reproducibility.

The presented lymphoma-on-chip model paves the way for developing and pre-clinical testing of new therapeutic strategies targeting the B-cell lymphoma microenvironment. Several cancer-on-chip studies including an endothelialized vessel have tested drug delivery in their systems. Two solid cancer-on-chip studies discussed above have successfully shown the effect of delivering blocking antibodies or small molecule inhibitors via the lymphatic vessel [33,37]. In a 3D microvascular DLBCL model, an antibody targeting tumor-associated macrophages was perfused through the endothelialized lumen which resulted in killing of the macrophages in the DLBCL tumor hydrogel [17]. Furthermore, the same model showed diffusion of a fluorescently-labelled antibody through the hydrogel, which was further increased by the presence of DLBCL tumor cells [17], suggesting that increased vessel permeability, which we also observe in our model, may be beneficial for drug delivery to the tumor. Furthermore, for (pre-)clinical implementation of our model, it will be crucial to incorporate patient samples. Most cancer-on-chip studies, including this study, use tumor cell lines as these are readily available, and primary patient samples are scarce. However, cell lines do not capture the heterogeneity found between patients and not all cells present in the tumor microenvironment can be sourced from cell lines. Especially for incorporation of T lymphocytes and testing of immunotherapies to improve the anti-tumor immune response, it will be required to use patient-matched cells in cancer-on-chip models [16]. In addition, we envision that the presented lymphoma-on-chip model can also be used for other B-cell malignancies originating in lymph nodes, such as follicular lymphoma and chronic lymphocytic leukemia, in which a pivotal role for stromal cells has been reported as well [73,74].

In conclusion, we generated a functional model of the human DLBCL microenvironment to study cellular interactions between tumor cells, FRCs and lymphatics. Our results using this model indicate that LNSCs, and specifically FRCs, promote DLBCL survival and migration. This lymphoma-on-chip model can be used for pre-clinical studies of potential therapeutic strategies to inhibit the tumor-supportive roles of LNSCs in DLBCL survival and dissemination. Furthermore, the model has the potential to be scaled-up for high-throughput testing enabling use in a (pre-)clinical setting to test drug response using a patient’s own tumor cells. As such, the presented lymphoma-on-chip model paves the way for personalized medicine options for DLBCL patients.

## 4 Methods

### 4.1 Tissue collection

Human LNs were obtained from donors from both sexes (Table S1) during liver transplant procedures performed at the Erasmus MC, Rotterdam, The Netherlands, in accordance to the Medical Ethical Committee (Medisch Ethische Toetsings Commissie; METC) of Erasmus MC (MEC-2014-060). All patients (liver transplant recipients) gave written informed consent to use their donor tissue. The LNs were resected along the hepatic artery and portal vein in the porta hepatis from donor livers. LNs were transported in Belzer University of Wisconsin (UW) cold storage solution (Bridge to Life Ltd., London, England, UK) and processed within 72 h of surgery.

### 4.2 Enzymatic digestion of human lymph nodes

Human LNs were enzymatically digested as published previously [19]. In short, LNs were minced and subsequently digested in 4 digestion cycles of 10 min with an enzyme mixture containing RPMI-1640 medium with 2.4 mg/mL Dispase II, 0.6 mg/mL Collagenase P and 0.3 mg/mL DNase I (all from Sigma-Aldrich, St. Louis, MO, USA). To prevent over-digestion and neutralize the digestion enzymes after each cycle, isolated cells were collected in ice-cold phosphate-buffered saline (PBS) supplemented with 2 % fetal calf serum (FCS) and 5 mM EDTA, and spun down at 300 g for 4 min at 4 °C. The cell pellet was re-suspended in 1 mL Dulbecco's Modified Eagle Medium (DMEM) (Gibco, Grand Island, NY, USA) supplemented with 10 % FCS, 2 % Penicillin/Streptomycin/Glutamine (PSG) and 1 % Insulin/Transferrin/Selenium (Gibco). After the last digestion cycle, all collected cells were filtered through a 100 μM filter and counted. The obtained human LN cell suspension was cultured to grow out FRCs and LECs.

### 4.3 Cell culture

To allow for an efficient and selective outgrowth of FRCs and LECs from LN cell suspensions, a seeding density of 1.25 × 10^6^ cell suspension per cm^2^ was used on culture flasks that were coated with 2 μg/cm^2^ collagen from calf skin (for FRCs) or 0.2 % gelatin diluted in PBS (for LECs) (both from Sigma-Aldrich). Culture media comprised of DMEM with 10 % FCS, 2 % PSG and 1 % Insulin/Transferrin/Selenium (for FRCs) or Endothelial Cell Growth Medium MV 2 (Ready-to-use; PromoCell GmbH via Merck, The Netherlands). After three days, lymphocytes were washed away with PBS to allow for optimal FRC and LEC growth. This process of washing away floating cells from the same flask was repeated twice per week during medium refreshments. Upon confluence, cells were harvest with PBS supplemented with 0.05 % trypsin and 5 mM EDTA for up to 5 min maximum. Trypsin was neutralised using culture medium (as above), and FRCs or LECs were passaged or collected for flow sorting (LECs) or use in experiments. FRCs and LECs were used up to and including passage 6 (FRCs) or passage 8 (LECs) for all individual experiments.

DLBCL cell line OCI-Ly18 (no. ACC 699) was purchased from DSMZ and cultured in IMDM (Gibco) with 10 % FCS and 2 % PSG. Cells were passaged twice a week at a cell concentration of 0.5 × 10^6^/mL and incubated at 37 °C, 5 % CO_2_. For use in lymphoma-on-chip experiments, 1 × 10^6^ DLBCL cells were labelled with 0.5 μM CellTracker™ Deep Red Dye (cat. no. C34565, Invitrogen via ThermoFisher Scientific) in serum-free IMDM for 20 min at 37 °C, 5 % CO_2_. Next, labelled cells were washed two times with IMDM supplemented with 10 % FCS and 2 % PSG, and brought to a concentration of 5 × 10^6^ cells/mL.

For static FRC-DLBCL co-cultures in hydrogel, 1.2 × 10^5^ FRCs and 0.3 × 10^5^ DLBCL single cells were gently mixed into a collagen/fibrinogen hydrogel [75]. The hydrogel/cell mixture was transferred on top of a 0.4 μm transwell in a 12-well plate, and allow to polymerize for 1.5 h at 37 °C and 5 % CO2. FRC medium was added both above and below the hydrogel, and was refreshed every 2–3 days. After 10 days, hydrogels were either fixed for immunohistochemistry.

### 4.4 Cell sorting of lymphatic endothelial cells

To obtain pure LEC cultures, we performed fluorescence-activated cell sorting (FACS) of cells cultured on gelatin. Cells were firstly washed with PBS and stained with a fixable viability dye (eBioscience™ Fixable Viability Dye eFluor™ 780, 1:3000, cat. no. 65-0865-14, Invitrogen via ThermoFisher Scientific) for 10 min at 4 °C. Next, cells were washed with PBS containing 0.1 % bovine serum albumin (BSA) (referred to as FACS buffer). Cells were then incubated with anti-CD31/PECAM-1-BV605 (1:100, cat. no. 303122, Biolegend) and anti-PDPN-Alexa Fluor 647 (1:50, cat. no. 337008, Biolegend) diluted in FACS buffer for 20 min at 4 °C. After staining, cells were washed two times with FACS buffer. All buffers and antibody solutions were filtered prior to use. Cells were sorted on FACSAria™ Fusion Flow Cytometer (BD Biosciences). Live CD31^+^ PDPN^+^ cells were collected in Endothelial Cell Growth Medium MV 2 (Ready-to-use) at RT and cultured in gelatin-coated flasks at 37 °C, 5 % CO_2_.

### 4.5 Fabrication of microfluidic chip

The mold design was made in Siemens NX (Siemens AG) and then transferred to PreForm software (Formlabs). A durable resin cartridge was inserted into a Low Force Stereolithography 3D printer (both from Formlabs), and the printing was run. When the print was complete, the platform was placed in Form-Wash (Formlabs) for 30 min in order to wash the uncured resin in isopropyl alcohol, and the resin then was fully cured in Form-Cure (Formlabs) for 1 h. The chip was made from a PDMS layer that was bonded to a glass slide using the following fabrication process. First, PDMS (Sylgard® 184 base with curing agent (both from Merck) at a 10:1 w/w ratio) was cast on the resin mold, degassed for 15 min, and cured at 65 °C. Then, the poured PDMS weight was measured and dosed upfront to obtain a final layer thickness of 5 mm. Next, the cured PDMS slabs were gently peeled off, and the PDMS edges were trimmed using a cutter. To punch in- and outlet access holes through the PDMS slab, 5 mm biopsy punchers (KAI biopsy punch 4560146922619) were used. Next, the channels were sealed by bonding of the PDMS layer to 25 mm × 75 mm glass slide (vwr). For this, the PDMS slab (features facing up) and the glass slide first were both exposed to 20 W air plasma for 30 s using a plasma asher (Emitech, K1050X). To achieve full bonding, the assembled complex underwent a thermal treatment at 65 °C for a minimum duration of 1 h.

### 4.6 Chip preparation and loading, and culture

The chips were autoclaved for sterility and all the next steps were conducted in a safety cabinet. The chips were located in a Petri dish (100 mm) for incubation, and a smaller Petri dish (35 mm) filled with Phosphate buffered solution (PBS) was placed next to the chip to increase the environmental humidity and to avoid excessive evaporation. The chamber of the chip was filled with 0.05 % w/v dopamine hydrochloride (poly-dopamine; Thermo Scientific Chemicals via Fisher Scientific) for 1 h at RT to enhance the hydrogel to PDMS attachment later. The polydopamine solution was aspirated out and the chamber was washed with PBS two times. All PBS was aspirated out, making sure that the chamber was empty. Steel acupuncture needles (160 μm diameter, Seirin) were introduced into the device through the lumen inlet of the chamber. The needle easily penetrated the PDMS to reach the lumen reservoir, and after needle extraction, the PDMS recovered with no leakage issues. Collagen type I (rat tail, 10 mg/mL, ibidi GmbH, Germany) solution was buffered with 10× PBS and titrated to a neutral pH with 0.1M NaOH, and brought to a final concentration of 5 mg/mL collagen I in cold FRC culture media. Per chip, DLBCL cells (0.5 × 10^6^) and FRCs (0.01 × 10^6^) were gently resuspended in the collagen mix, and 100 μl of collagen-cell solution was transferred to the chamber of the microfluidic device and polymerized for 45 min at 37 °C, 5 % CO_2_. After polymerization, needles were gently extracted to create a hollow lumen. Next, 10 μl of LECs (4 × 10^6^/mL) were pipetted into the lumens and incubated for 1 h at 37 °C, 5 % CO_2_ to facilitate attachment to the luminal base. After incubation, the reservoirs of the lumen were filled with LECs medium and lateral hydrogel reservoirs with FRC medium. Media was changed twice a day on working days. For the lumen, the LEC medium was added only from one reservoir, flowing through the lumen to the other reservoirs due to hydrostatic pressure. Chips were cultured for 7 days at 37 °C, 5 % CO_2_.

### 4.7 Dextran permeability assay and analysis

Solute transport from the vessel lumen was measured by dextran diffusion. Dextran Texas-Red (70 kDa, cat. no. D1864, Invitrogen via ThermoFisher Scientific) solution was prepared to a stock concentration of 25 mg/mL in PBS. The working concentration of 25 μg/mL was freshly prepared by further dilution in LEC media. For the permeability assays, 50 μl of dextran solution was added to one of the vessel reservoirs and time-lapse imaging was performed on a Nikon Ti2 widefield fluorescence microscope. Solute transport was measured over 15 min per vessel. Permeability coefficients were calculated using following equation [76]:

$$P=\left(\frac{1}{{\Delta I}_{0}}\right)\left[\left({TI}_{f}-{TI}_{0}\right)/\left(t_{f}-t_{0}\right)\right]\left(D/4\right)$$

where ΔI_0_ is the initial step increase in fluorescence intensity across the lumen wall, TI_0_ is the total initial fluorescence intensity along the line starting from the edge of the vessel and going outwards at the beginning of the assay, TI_f_ is the total fluorescence intensity along the line starting from the edge of the vessel and going outwards at 15 min, t_0_ is the initial time point, t_f_ is the final time point of 15 min, and D is vessel diameter. For calculating ΔI_0_, it is assumed that the concentration difference of dextran across the lumen wall at the beginning of the experiments equals the solute concentration in the lumen.

### 4.8 Transwell migration assay

2.5 × 10^4^ FRCs or LECs per well were seeded in 24-well plate and incubated at 37 °C, 5 % CO_2_. After 48 h, 0.5 × 10^6^ DLBCL cells per condition were seeded in 100uL collagen hydrogel (2.5 mg/mL; recipe as above) on top of 6.5 mm Transwell® with 5.0 μm Pore Polycarbonate Membrane Insert (Corning®, NY, USA) and hydrogels were polymerized for 45min at 37 °C, 5 % CO_2_. Next, transwells were placed on top of the FRCs or LECs or their respective media as control, and 100uL IMDM with 10 % FCS and 2 % PSG was placed on top of the hydrogels. DLBCL cells were allowed to migrate for 5 days, after which migrated cells in the bottom compartment of the well were collected, washed with PBS, stained with a fixable viability dye (eBioscience™ Fixable Viability Dye eFluor™ 780, 1:3000, cat. no. 65-0865-14, Invitrogen via ThermoFisher Scientific) for 10 min at 4 °C, and acquired on Aurora 5-laser Flow Cytometer (Cytek, Amsterdam, The Netherlands) to analyze live cell count. Supernatants of migrated cells were collected and stored at −20 °C.

### 4.9 Cytokine bead array

Supernatants were harvested, spun down to remove cell debris and stored at −20 °C until analyzed for the presence of cytokines and chemokines using cytokine bead arrays (Human Custom LEGENDplex panel; BioLegend), according to the manufacturer’s instructions. Acquisition was performed on AttuneNxT (ThermoFisher, Waltham, MA, USA) and protein concentrations were determined using the LEGENDplex Data Analysis Software Suite (BioLegend).

### 4.10 ELISA

Supernatants were harvested, spun down to remove cell debris and stored at −20 °C until IL-6 levels (transwell samples 1:100 dilution, lymphoma-on-chip samples undiluted) were measured using Human IL-6 Uncoated ELISA (cat. no. 88–7066, Invitrogen via ThermoFisher Scientific, The Netherlands), according to the manufacturer’s instructions. Absorbance was measured at 450 nm using BioTek 800 TS Absorbance Reader (Agilent, Santa Clara, CA, USA).

### 4.11 Histology

Paraffin sections (5 μm) of non-malignant (reactive) LNs and DLBCL tumors were obtained from the Pathology Tissue Research facility of Amsterdam UMC. Collagen hydrogels from FRC-DLBCL co-cultures were fixed in 4 % PFA. For paraffin embedding, samples were dehydrated, embedded in paraffin and cut at 5 μm sections for immunofluorescent (IF) analysis. Sections were deparaffinised and immersed in Tris-EDTA buffer (pH 9.0) for 10 min at 100 °C for antigen retrieval, followed by slowly cooling to RT. Sections were then washed with PBS and blocked for 20 min with 1 % bovine serum albumin and 10 % normal goat serum in PBS. Next, tissue sections were stained with anti-Vimentin-Alexa Fluor 488 (1:200, cat. no. 677809, Biolegend) for 1 h, washed three times with PBS for 5 min and stained with DAPI (5 μg/mL, D1306, Invitrogen via ThermoFisher Scientific) for 10 min. After three washes with PBS for 5 min, sections were mounted with Fluoromount-G® (cat. no. 0100-01, SouthernBiotech, Birmingham, AL, USA) and stored at RT until acquisition. Images were acquired on Zeiss Axio Imager D2 (Zeiss, Jena, Germany) and processed using Fiji software [77].

### 4.12 Time-lapse imaging

Time-lapse images were acquired on Nikon AX R confocal microscope (Nikon, Amstelveen, The Netherlands). Number of CellTracker Deep Red-labelled DLBCL cells in the hydrogel and distance of CellTracker Deep Red-labelled DLBCL cells to the LEC vessel was analyzed using Nikon Imaging Software. Migration of CellTracker Deep Red-labelled DLBCL cells were analyzed using Imaris software (version 9 or higher; Oxford Instruments, Abingdon, UK). First, we used the spots function (settings: XY = 5 μm, Z = 20 μm) to identify CellTracker Deep Red labelled cells with a diameter of 5 μm or more to exclude background signal. Next, we ran the Tracking plugin to calculate the track straightness of each spot, i.e. of each CellTracker Deep Red labelled cell.

### 4.13 Immunofluorescence staining & imaging

Chips were washed two times with PBS, fixed by incubating in 4 % paraformaldehyde (Aqueous Solution, EM Grade; Electron Microscopy Sciences via Aurion, Wageningen, The Netherlands) for 10 min at RT, again washed two times with PBS and stored at 4 °C until staining was continued. Permeabilize using 0.2 % Triton X-100 in PBS for 15 min at RT, followed by blocking with 1 % bovine serum albumin/0.2 % Triton X-100 in PBS for 1 h at RT on an orbital shaker (150 rpm). Next, lumens of chips were incubated with goat-anti-human VE-cadherin (1:100; cat. no. AF938, R&D Systems) or mouse-anti-human CD31 (1:100; M0823, DAKO) overnight at 4 °C, shaking. Hydrogel reservoirs were filled with block buffer. The next day, chips were washed three times with PBS for 10 min at RT, shaking, followed by incubation with respectively Alexa Fluor 555-labelled donkey-anti-goat or goat-anti-mouse antibodies (1:400; Invitrogen, via ThermoFisher Scientific) for 1 h at RT, shaking. Next, chips were washed three times with PBS for 10 min at RT, shaking, followed by incubation with mouse-anti-human Vimentin-Alexa Fluor 488 (1:200; cat. no. 677809, Biolegend) and DAPI (0.5 μg/mL, D1306, Invitrogen via ThermoFisher Scientific). Finally, chips were washed three times with PBS for 10 min at RT, shaking, and stored at 4 °C until imaging. Images were acquired on Nikon AX R confocal microscope (Nikon, Amstelveen, The Netherlands) and processed using Fiji software [77]. 3D rendering was performed in Imaris software (version 9 or higher; Oxford Instruments, Abingdon, UK).

### 4.14 Statistics

Statistical analysis was performed using two-way ANOVA with Tukey’s multiple comparisons test, or in case of non-Gaussian distribution, Kruskal–Wallis test with Dunn’s multiple comparisons test in GraphPad Prism 9 software (GraphPad Software Inc., San Diego, CA, USA). Differences were considered to be significant when *p* < 0.05.

## CRediT authorship contribution statement

**Mohammad Jouybar:** Writing – review & editing, Writing – original draft, Visualization, Validation, Resources, Methodology, Investigation, Conceptualization. **Aleksandra M. Mikula:** Investigation. **Nanouk Zuidmeer:** Investigation. **Tanja Konijn:** Validation, Investigation. **A. Vera de Jonge:** Writing – review & editing, Resources. **Henk P. Roest:** Resources. **Tuna Mutis:** Resources. **Luc J.W. van der Laan:** Writing – review & editing, Resources. **Reina E. Mebius:** Writing – review & editing, Resources. **Jaap M.J. den Toonder:** Writing – review & editing, Supervision, Resources, Funding acquisition. **Charlotte M. de Winde:** Writing – review & editing, Writing – original draft, Visualization, Validation, Supervision, Project administration, Methodology, Investigation, Funding acquisition, Conceptualization.

## Declaration of competing interest

The authors declare that they have no known competing financial interests or personal relationships that could have appeared to influence the work reported in this paper.

## Figures and Tables

**figs1:**
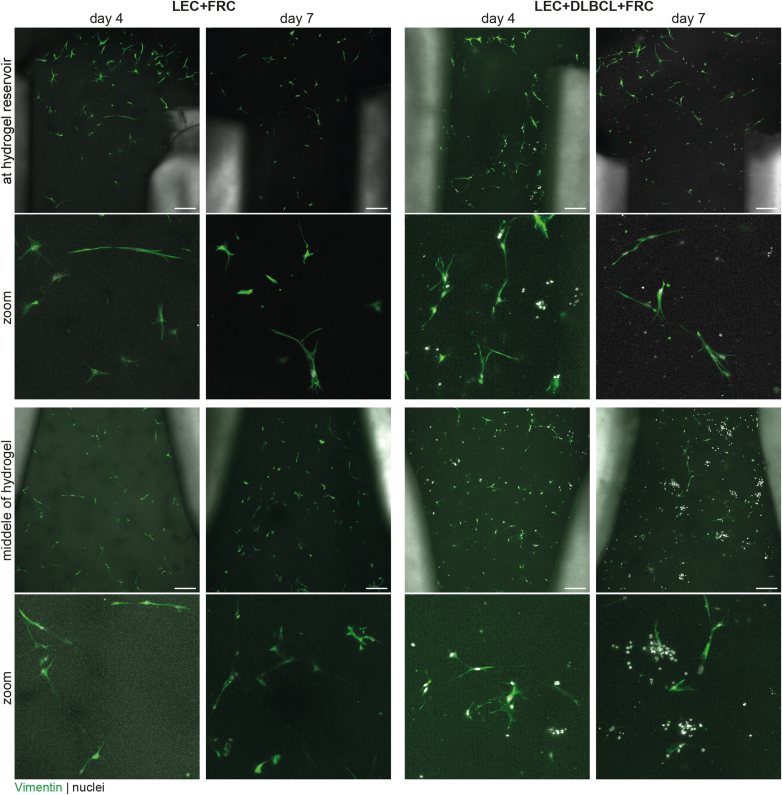


## Data Availability

Data will be made available on request.
