# Supplementary material for: Lymphoma-on-chip model reveals that lymph node stromal cells promote diffuse large B-cell lymphoma survival and migration
Source: Mater Today Bio. 2025 Feb 7;31:101544. doi: 10.1016/j.mtbio.2025.101544 (PMC11889632; doi:10.1016/j.mtbio.2025.101544)
Supplement: Multimedia component 1 [file mmc1.docx]

**Supplemental information**

**Supplementary movie 1.** 3D rendering of lymphoma-on-chip model stained for CD31 (green) and Vimentin (magenta). DLBCL cells (cyan) are labelled with CellTracker Deep Red prior to seeding in collagen hydrogel.

**Supplementary movie 2.** Time-lapse imaging of CellTracker Deep Red-labelled DLBCL cells (red) in collagen-hydrogel without FRCs. Tracks of individual CellTracker Deep Red-labelled DLBCL cells (red) are shown in movie 2B.

**Supplementary movie 3.** Time-lapse imaging of CellTracker Deep Red-labelled DLBCL cells in collagen-hydrogel with FRCs. Tracks of individual CellTracker Deep Red-labelled DLBCL cells (red) are shown in movie 3B.

**Figure S1.** Immunofluorescence staining of Vimentin (labelling FRCs; green) in a chip with LEC+FRC (left) or LEC+DLBCL+FRC (right) after 4 and 7 days of culture. Top panel shows the area close to the hydrogel reservoir, and bottom panel the area in the middle of the hydrogel. Nuclei are shown in white. Scale bar is 200 microns.

**Table S1. Human lymph node donor characteristics**, related to Figures 1, 2, 3 & 4.

F: Female, M: Male, DBD: Donation after Brain Death, DCD: Donation after Circulatory Death

| **#** | **Sex** | **Age** | **Donor type** | **Used in Figure** |
| --- | --- | --- | --- | --- |
| 1 | M | 75 | DBD | 1 |
| 2 | F | 39 | DCD | 2,3,4 |
| 3 | M | 56 | DBD | 3,4 |
| 4 | F | 62 | DBD | 1,2,3,4 |
| 5 | F | 53 | DCD | 2,3,4 |
